# Correlation between perioperative parecoxib use and postoperative acute kidney injury in patients undergoing radical mastectomy: a retrospective cohort analysis

**DOI:** 10.1186/s12871-022-01688-4

**Published:** 2022-05-20

**Authors:** Nan Xu, Ke Pang, Sihua Qi, Hongmei Wang

**Affiliations:** 1 The Department of Anesthesiology, The Fourth Affiliated Hospital of Harbin Medical University, 37 Yiyuan Street, Nangang District, Harbin, Heilongjiang China; 2 The Department of Anesthesiology, The Third Xiangya Hospital of Central South University, 138 Tongzipo Road, Yuelv District, Changsha, Hunan China; 3 The Department of Pain, The Fourth Affiliated Hospital of Harbin Medical University, 37 Yiyuan Street, Nangang District, Harbin, Heilongjiang China

**Keywords:** Acute kidney injury, Parecoxib, Mastectomy

## Abstract

**Background:**

Non-steroidal anti-inflammatory drugs (NSAIDs) are among the most widely prescribed drugs worldwide. However, the effect of NSAIDS on postoperative renal function is still unclear. Few studies have assessed the effects of parecoxib on renal function. Our aim is to investigate a correlation between parecoxib and the presence or absence of AKI postoperatively after a breast cancer surgery operation.

**Methods:**

This was a retrospective cohort study that we performed on our hospitalized database. From January 2012 to August 2021, 3542 female patients undergoing radical mastectomy were enrolled, all data including the patients' information and laboratory results were obtained from electronic medical system. The main outcome was the incidence of AKI postoperatively. AKI was defined in accordance with the KDIGO criteria. Study groups were treated with or without parecoxib. Univariable and multivariable logistic regression analyses were performed.

**Results:**

In our study, about 5.76% experienced AKI. The incidence rate of postoperative AKI (3.49%) within 7 days in the parecoxib group was lower than that in the control group (6.00%, *P* = 0.05). Compared to the control group, the AKI’s incidence was reduced by 49% (OR = 0.46; 95%CI 0.27–0.97) in parecoxib group in multivariable logistic regression analysis. There was a reduction in the incidence of postoperative AKI in other three subgroups: preoperative eGFR < 90 mL/min·1.73/m2 (OR = 0.52; 95%CI 0.27–0.97), blood loss < 1000 ml (OR = 0.48; 95%CI 0.24–0.96) and non-diabetes (OR = 0.51; 95%CI 0.26–0.98).

**Conclusions:**

Parecoxib was associated with incidence of postoperative acute kidney injury.

## Background

Acute kidney injury (AKI) is a common postoperative complication among surgical patients [1, 2]. Previous researches suggested that AKI was an independent predictors of increasing length and expenses of hospitalization, and increased mortality in patients with surgery [3, 4]. AKI was a well-recognized complication after cardiac surgery with an incidence of up to 40% [5]. Previous study demonstrated that the postoperative AKI incidence reached to 6.8% in non-cardiac surgery [6]. Besides, among those patients with good physical conditions (low ASA grade) who undergone non-cardiac surgery, the incidence of postoperative AKI could also reach to 6% [7, 8].

There were many risk factors related to in hospital AKI. The most common cause of hospital-acquired AKI is whole-body hypoperfusion, which is also independently associated with perioperative AKI [9]. Up to 60% of cases with sepsis are complicated with AKI, and approximately half of AKI cases are related to sepsis [10]. Besides, AKI was an acute worsening of renal function often associated with the use of contrast agents [11]. Meanwhile, AKI is reported to be a heterogeneous clinical syndrome, with nephrotoxic drugs accounting for 15% of AKI cases [12]. After all, the multifactorial nature of AKI and the numerous nephrotoxic drugs potentially delivered to patients make it very challenging to delineate attributable risk of AKI to specific drugs [13]. However, appropriate drugs that suppress inflammation and oxidative stress might be favorable in treating sepsis-associated AKI [14].

Non-steroidal anti-inflammatory drugs (NSAIDs) are among the most commonly used medicines in the world. Parecoxib was a kind of selective NSAIDs. Tang discovered that the AKI after the non-cardiac surgery was potential associated with parecoxib [15]. Besides, previous studies believed that COX-2 can protect the kidney from ischemia–reperfusion injury in the rats [16]. Therefore, selective COX-2 inhibitors may aggravate renal injury [17]. Above all, the effect of NSAIDS on postoperative renal function is still unclear. The objective of the study was as follows: To evaluate the correlation between postoperative AKI and the parecoxib in the patients undergoing the first surgery for primary breast cancer.

## Methods

**Fig. 1:**
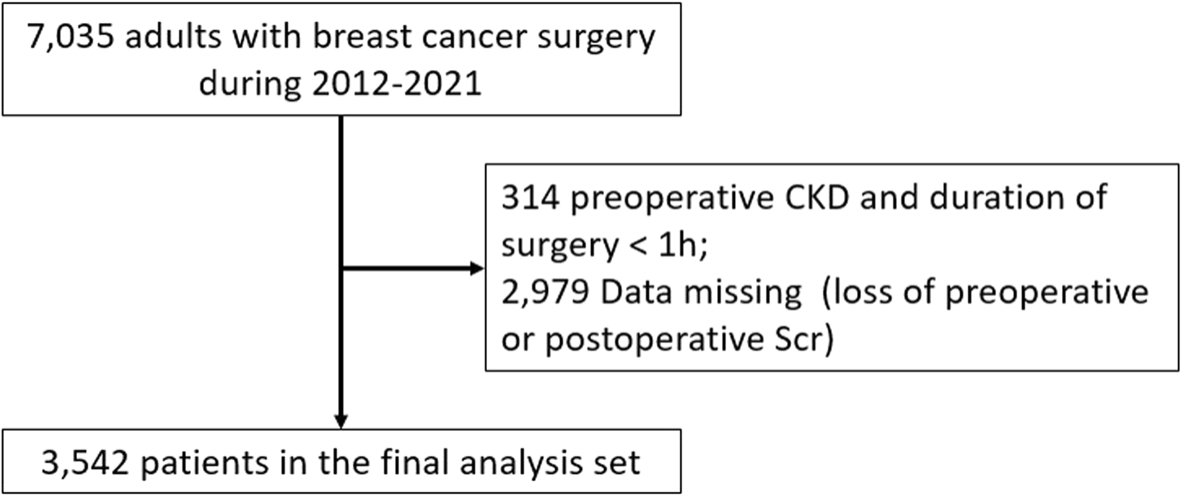
Enrollment of patients undergoing breast cancer surgery

This was a retrospective cohort study in single hospital between January 1^st^ 2012 and August 31^st^ 2021. The inclusion criteria were as following: (1) patients > 17 years old (2) underwent primary surgical treatment of breast cancer. And the exclusion criteria were as following: (1) surgery duration less than 1 h, (2) preoperative or postoperative creatinine were missing, or (3) preoperative combined chronic kidney disease (CKD), defined as estimate glomerular filtration rate (eGFR) < 60 mL/min/1.73/ m^2^, ≥ 3 months). Fig. 1 showed the details.

### Data collection

The following data was collected: (1) demographic information, including age, sex and body mass index(BMI); (2) individual history including preoperative complications and medication history; (3) laboratory data including serum creatinine and eGFR calculated using the CKD Epidemiology Collaboration formula; (4) intraoperative data including emergency, surgical grade, operative time, anesthesia method, ASA grade, amount of fluid infusion and out, intraoperative erythrocyte transfusion volume, amount of blood loss, intraoperative hypotension and vasoactive drugs and (5) postoperative outcomes such as the occurrence of AKI, admission to intensive care unit (ICU) and mortality. The missing values will be imputed by multiple imputation.

### Primary outcome and definitions

Study groups were treated with or without parecoxib. Parecoxib group was only administered (40 mg or 80 mg) for one time during the induction period at the discretion of the trained anesthesiology. Whether to use the parecoxib or not was based on the doctor’s preference. Parecoxib doses larger than 80 mg were not included because the routine dose is not more than 80 mg/day based on the drug instructions. Intraoperative hypotension was defined as mean arterial pressure < 65 mmHg for a duration of at least 5 min.

Postoperative AKI was defined by Kidney Disease Improving Global Outcomes classification (KDIGO) without urine output criteria [18]. Increase in serum creatinine by > 1.5-fold from preoperative baseline within 7 days or increase in serum creatinine ≥ 0.3 mg dL^−1^ within 48 h. The major outcome event was whether the postoperative AKI occurs or not.

### Statistical analysis

Sample size was determined setting alpha = 0.05 and power = 0.80. Based on an estimated AKI incidence of 6.3% in control group and 3.6% in parecoxib group, we would need to include approximately 2004 patients. Empowerstats (http://www.empowerstats.com), R statistical software package and SAS statistical software were used for data analysis. Continuous variables are expressed by mean (SD), and classification variables were expressed in frequency and percentage and were analyzed using the Chi-squared test or Fisher exact test. Continuous variables between subgroups were compared by using Kruskal–Wallis test. Logistic stepwise regression was used in multivariate analysis. Bilateral test was used in all statistical analysis. The results of categorical variables were expressed expressed as OR or ‘beta’ and 95% CI; significance was accepted at a *P* value of less than 0.05.

## Results

### Parecoxib

Overall, the parecoxib was used by 9.71% (344/3542) of the patients before the end of the surgery. The percentage of AKI in the parecoxib group (12/344, 3.49%) was lower than that control group (192/3198, 6.00%). No statistical differences between the two groups with respect to age, eGFR, alcoholism, anemia, diabetes mellitus, preoperative ACEI, ARB, diuretics, ASA grade, intraoperative erythrocyte transfusion, and intraoperative hypotension were observed (Tables 1 and 2).

**Table 1:** Baseline demographic data of study population group by parecoxib

Clinical features	Control Group(*n* = 3198)	Parecoxib Group(*n* = 344)	*P*
Age (year)	45.30 ± 9.53	45.15 ± 9.81	0.921
BMI	22.68 ± 6.35	21.96 ± 2.73	0.049
eGFR	98.85 ± 21.02	100.14 ± 21.55	0.270
Alcohol consumption	39 (1.22%)	3 (0.87%)	0.572
Anemia	648 (20.26%)	59 (17.15%)	0.170
Hypertension	714 (22.33%)	57 (16.57%)	0.014
Diabetes mellitus	169 (5.28%)	14 (4.07%)	0.333
ACEI	57 (1.78%)	5 (1.45%)	0.659
ARB	34 (1.06%)	2 (0.58%)	0.397
CCB	426 (13.32%)	28 (8.14%)	0.006
Diuretics	35 (1.09%)	4 (1.16%)	0.908
ASA grade			0.932
I–II	2572 (80.43%)	276 (80.23%)	
III–V	626 (19.57%)	68 (19.77%)	
Operative time (min)			< 0.001
≤ 60	442 (13.82%)	27 (7.85%)	
61–120	841 (26.30%)	75 (21.80%)	
121–180	812 (25.39%)	98 (28.49%)	
> 180	1103 (34.49%)	144 (41.86%)	
Intraoperative erythrocyte Transfusion, mL (%)			0.396
< 100	2465 (77.08%)	315 (77.91%)	
100–600	355 (11.10%)	29 (8.43%)	
601–1000	173 (5.41%)	22 (6.40%)	
> 1000	205 (6.41%)	25 (7.27%)	
Intraoperative hemorrhage, mL (%)			0.004
< 100	1030 (32.21%)	81 (23.55%)	
100–600	1728 (54.03%)	205 (59.59%)	
601–1000	236 (7.38%)	26 (7.56%)	
> 1000	204 (6.38%)	32 (9.30%)	
Intraoperative hypotension	345 (10.79%)	47 (13.66%)	0.216
In fluid amount (10 mL/24 h)	937.50 (625.00–1369.79)	1083.33 (666.67–1500.00)	< 0.001
Out fluid amount (10 mL/24 h)	333.33 (145.83–541.67)	291.67 (125.00–479.17)	0.004

*AKI* Acute Kidney Injury, *BMI* Body Mass Index, *eGFR* estimated Glomerular Filtration Rate, *ACEI* Angiotensin-converting Enzyme Inhibitors, *ARB* Angiotensin Receptor Blockers, *CCB* Calcium-channel Blockers, *ASA* American Society of Anesthesiologists. Data are expressed as number of patients (%) or Mean ± SD

**Table 2:** Incident of postoperative AKI in different dose paracoxib

	Paracoxib(0 mg)	Parecoxib(40 mg or 80 mg)	
AKI	192 (6.00%)	12 (3.49%)	0.051
AKI.Stages			0.225
0	3006 (94.00%)	332 (96.51%)	
1	140 (4.38%)	8 (2.33%)	
2	25 (0.78%)	1 (0.29%)	
3	27 (0.84%)	3 (0.87%)	

*AKI* Acute Kidney Injury. Data are expressed as number of patients (%)

### Acute kidney injury

In total, 3542 patients were included in this study. AKI incidence was 5.76% (204/3542), with 3.92% (8/204) admission into ICU and mortality rate of 1.96% (4/204). No significant differences in age, BMI, alcohol consumption, the use of ACEI, ARB, intraoperative hemorrhage and admission into ICU were observed between groups. Baseline demographics between groups are shown in Table 3.

**Table 3:** Baseline demographic data of study population group by AKI

Clinical features	Without AKI(*n* = 3338)	With AKI(*n* = 204)	*P*
Age (years)	45.29 ± 9.56	45.06 ± 9.59	0.63
BMI	22.64 ± 6.22	22.18 ± 3.46	0.15
eGFR	100.28 ± 18.88	77.61 ± 37.41	< 0.001
Alcohol consumption	40 (1.20%)	2 (0.98%)	0.78
Anemia	649 (19.44%)	58 (28.43%)	0.002
Hypertension	705 (21.12%)	66 (32.35%)	< 0.001
Diabetes mellitus	166 (4.97%)	17 (8.33%)	0.03
ACEI	58 (1.74%)	4 (1.96%)	0.81
ARB	33 (0.99%)	3 (1.47%)	0.50
CCB	417 (12.44%)	37 (18.14%)	0.02
Diuretics	33 (0.99%)	6 (2.94%)	0.01
ASA grade			< 0.001
I–II	2704 (81.01%)	144 (70.59%)	
III–V	634 (18.99%)	60 (29.41%)	
Operative time (min)			0.004
≤ 60	446 (13.36%)	23 (11.27%)	
61–120	882 (26.42%)	34 (16.67%)	
121–180	852 (25.52%)	58 (28.43%)	
> 180	1158 (34.69%)	89 (43.63%)	
Intraoperative erythrocyte Transfusion, mL (%)			0.004
< 100	2596 (77.77%)	137 (67.16%)	
100–600	353 (10.58%)	31 (15.20%)	
601–1000	181 (5.42%)	14 (6.86%)	
> 1000	208 (6.23%)	22 (10.78%)	
Intraoperative Hemorrhage, mL (%)			0.12
< 100	1054 (31. 58%)	57 (27.94%)	
100–600	1824 (54.64%)	109 (53.43%)	
601–1000	240 (7.19%)	22 (10.78%)	
> 1000	220 (6.59%)	16 (7.84%)	
In fluid amount (10 mL/24 h)	937.50 (625.00–1375.00)	1135.42 (729.17–1526.04)	< 0.001
Out fluids amount (10 mL/24 h)	312.50(145.83–541.67)	333.33 (187.50–625.00)	0.02
Intraoperative hypotension	363(10.87%)	29(14.21%)	0.005
Parecoxib	332 (9.95%)	12 (5.88%)	0.05
Admission to ICU	90 (2.70%)	8 (3.92%)	0.30
Death	7 (0.21%)	4 (1.96%)	< 0.001

*AKI* Acute Kidney Injury, *BMI* Body Mass Index, *eGFR* estimated Glomerular Filtration Rate, *ACEI* Angiotensin-converting Enzyme Inhibitors, *ARB* Angiotensin Receptor Blockers, *CCB* Calcium-channel Blockers, *ASA* American Society of Anesthesiologists, Intraoperative hypotension: MAP < 60 mmHg, *ICU* Intensive Care Unit. Data are expressed as number of patients (%) or Mean ± SD

### Univariable analysis

**Fig. 2:**
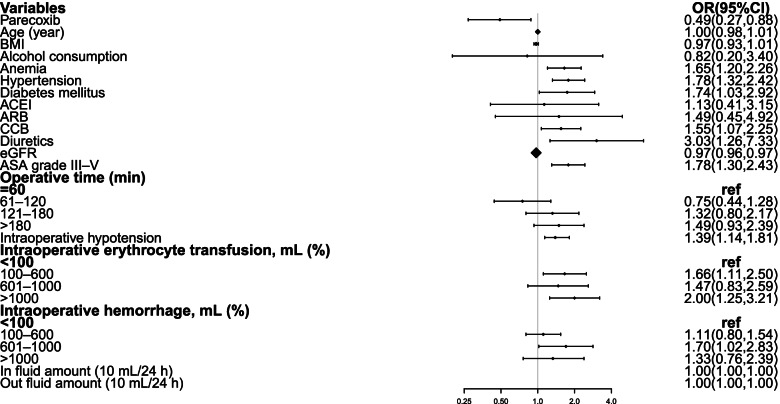
Univariable analysis of AKI

Univariable analysis was shown in the Fig. 2. By the univariable analysis, the use of parecoxib (OR 0.49; 95% CI 0.27–0.88, *P* = 0.017), anemia (OR 1.65; 95%CI 1.20–2.26, *P* = 0.002), hypertension(OR 1.78; 95% CI 1.32–2.42, *P* < 0.001), diabetes (OR 1.74; 95% CI 1.03–2.92, *P* = 0.038), the use of CCB ( OR 1.55; 95% CI 1.07–2.25, *P* = 0.020), diuretics (OR 3.03; 95% CI 1.26–7.33, *P* = 0.013), eGFR (OR 0.97; 95% CI 0.96 to 0.97, *P* < 0.001), ASA grade III–V ( OR 1.78; 95% CI 1.30–2.43, *P* < 0.001), intraoperative erythrocyte transfusion 100-600 ml (OR 1.66; 95% CI 1.11–2.50, *P* = 0.014), intraoperative erythrocyte transfusion > 1000 ml (OR 2.00; 95% CI 1.25–3.21, *P* = 0.004), intraoperative hemorrhage between 100-600 ml (OR 1.70; 95% CI 1.02–2.83, *P* = 0.043) and in fluid amount (OR 1.00; 95% CI 1.00–1.00, *P* < 0.001) were associated with the postoperative AKI independently. Age, BMI, alcohol consumption, the use of ACEI, ARBs, and operative duration were not correlated with AKI.

### Multivariable regression analysis

Table 4 showed the results of Multivariable regression analysis. Parecoxib was independently associated with postoperative AKI. Besides, parecoxib might have moderate protective effects on postoperative renal function in the subgroups of eGFR < 90, blood loss < 1000 ml, and non-diabetes groups. For all the three subgroups, the administration of parecoxib leaded to a decreasingly incidence of postoperative AKI.

**Table 4:** Multivariable regression analysis of postoperative AKI associated with parecoxib

AKI	Control	Parecoxib(40 or 80 mg)		
		Model 1	Model 2	Model 3
All cases	1	0.49 (0.27, 0.88) 0.017	0.48 (0.26, 0.87) 0.016	0.46 (0.24, 0.86) 0.015
EGFR < 90	1	0.58 (0.32, 1.04) 0.069	0.56 (0.31, 1.02) 0.058	0.52 (0.27, 0.97) 0.041
Blood loss < 1000 ml	1	0.52 (0.27, 1.00) 0.051	0.51 (0.27, 0.98) 0.045	0.48 (0.24, 0.96) 0.037
Non-diabetes	1	0.56 (0.30, 1.04) 0.066	0.54 (0.29, 1.01) 0.053	0.51 (0.26, 0.98) 0.045
AKI Stages	1	-0.04 (-0.07, -0.00) 0.030	-0.04 (-0.07, -0.00) 0.028	-0.04 (-0.07, -0.01) 0.013

Model 1: Non-adjusted

Model 2: Adjusted for age, BMI, alcohol consumption, anemia, hypertension, diabetes mellitus, ACEI, CCB, diuretics, ASA, anesthesia method, in fluids, out fluids, transfusion, and hemorrhage

Model 3: Adjusted for model 2 plus ARB, preoperative eGFR, anesthesia method, operative time and intraoperative hypotension

## Discussion

There are so many factors related to the post-operative AKI (Fig. 2). ASA grade were related to the post-operative AKI which was similar to the previous researches [7]. The Fig. 1 suggested that intraoperative hypotension (MAP < 60 mmHg) was a risk factor to AKI, too [19]. Besides, according to the previous studies [20, 21], preoperative eGFR was one of the most important factors to AKI. The analysis suggested that preoperative eGFR was an independent risk factor in our study.

Over the past years, many researches about the NSAIDs and renal function have been reported. A previous meta-analysis suggested that the AKI was associated to NSAIDs exposure, especially in the elderly patients [22]. And another meta-analysis demonstrated selective COX-2 inhibitors was related with the increasing risk of AKI [23]. However, a study revealed that there were no statistical differences in kidney function between the groups with and without NSAIDs among elderly patients [24]. Even more, a prospective and multicenter study by STARSurg showed that NSAIDs in the postoperative was safer for the patients undergoing gastrointestinal surgery. And the low dose of flurbiprofen axetil (50-100 mg) decrease the rate of AKI after non-cardiac surgery [25, 26].

In our study, 5.76% patients experienced postoperative AKI after breast cancer surgery which was in the scope mentioned in previous studies (1.0–31.0%) [27–29]. The incidence of AKI in cancer patients was high, which was closely related to their cachexia or renal injury drugs [30]. And another study showed that non-selective NSAIDs (acetaminophen) was not related to the AKI among the patients undergoing surgery for renal carcinoma [31]. However, administration of parecoxib nearly reached statistical significance in our study (Table 2). One possible explanation might be that breast tumor growth have elevated the level of inflammation [32] and reduced the potential renoprotetive effect of parecoxib. Furthermore, parecoxib showed its potential renoprotective effect in the multivariable analysis (OR = 0.46, 95%CI = 0.24–0.86) in Table 4. Furthermore, the results revealed that parecoxib may still has a stable effect on the partial patients receiving breast cancer surgery as following: eGFR < 90 mL/min·1.73/m^2^, blood loss < 1000 ml and non-diabetes, which represented preoperative glomerular perfusion and filtration disorder, relatively good cardiovascular status and perfusion. Overall, parecoxib (40 or 80 mg) has a potential protective effect on postoperative AKI.

The mechanism by which parecoxib protects the patient against postoperative AKI is not clear. But it maybe correlated with decreasing the postoperative kidney inflammatory reaction [33]. High-dose flurbiprofen axetil may destroy the inflammatory balance in the tubules [25]. Besides the clinical studies above, an animal experiment by Takaku indicated that a single dose of parecoxib could reduce the inflammation and tubular renal injury in the rats model of ischemic tubular necrotizing nephritis caused by acute hemorrhagic shock [34].

A limitation of the present study was its retrospective nature, and further research involving multi-center, prospective studies is required. And, we only chose the patients above 18 years who received the first breast cancer surgery, and the scope of application of the conclusion was relatively narrow.

## Conclusions

Parecoxib (40 or 80 mg) may correlate with relatively low risk of postoperative AKI, in adult patients undergoing breast cancer surgery. Further prospective, multiple centers research are needed to confirm the protective effects on postoperative AKI.

## Data Availability

The analyzed data sets generated during the study are available from the corresponding author on reasonable request.
